# Gauging Public Health Literacy needs through Google Trends: the COVID-19 vaccination example

**DOI:** 10.1093/eurpub/ckac131.176

**Published:** 2022-10-25

**Authors:** J Rachadell, R Vareda, A Santos

**Affiliations:** 1 Public Health Unit, ACES Lisboa Ocidental e Oeiras, Oeiras, Portugal; 2 EUPHA-DH; 3 Institute for Evidence Based Health, Lisbon, Portugal; 4 Public Health Unit, ACES Lisboa Norte, Lisbon, Portugal; 5 Public Health Unit, ACES Almada-Seixal, Almada, Portugal

## Abstract

**Background:**

The rollout of the COVID-19 vaccination program in Portugal was one of the most successful in the world. However, there was still a challenge for Public Health services to determine the health literacy needs of the population in the context of a new vaccine and the uncertainty of the pandemic. The goal of this study was to evaluate the usefulness of Google Trends data to gauge the health literacy needs of the general population during the pandemic.

**Methods:**

A Google Trends search was performed for Portugal including 5 topics related to health literacy needs of the population (“vaccination”, “scheduling”, “isolation”, “booster dose” and “vaccination certificate” ) between the 28th of december 2019 and the 10th of march 2022. The variation shows the relative popularity of each term referring to the total number of Google searches during that period in a normalized scale of 0-100. The variation was compared to the number of doses administered daily in Portugal and key moments of the vaccination campaign as defined by the Directorate-General for Health in Portugal.

**Results:**

The terms “vaccination” and “isolation” had a steady rise in popularity from December 2019 to January 2021. The term “vaccination” was the most popular search term with peak popularity in July 2021 and a downward trend followed by an ascent to a lower peak in popularity in January 2022. The terms “scheduling” and “vaccination certificate” both followed a similar pattern, though at lower popularity levels. The term “isolation” was low on popularity since February 2021, with a significant rise and peak in January 2022. This variation relates to key dates during the Portuguese vaccination campaign.

**Conclusions:**

Google Trends data seems to correlate with key events during the Portuguese COVID-19 vaccination campaign. That data might be incorporated in the planning framework of health literacy activities for national and local Public Health services.

**Key messages:**

• Google Trends might be an important source of information for public health teams.

• There is a need for further research into how Google Trends data can be incorporated with other sources of information to inform health literacy activities.

